# Comparing the Effectiveness of Neurofeedback and Transcranial Direct Current Stimulation on Sleep Quality of Patients With Migraine

**DOI:** 10.32598/BCN.10.6.651.3

**Published:** 2019-11-01

**Authors:** Zahra Kosari, Mohsen Dadashi, Mahdi Maghbouli, Hossin Mostafavi

**Affiliations:** 1. Department of Clinical Psychology, Faculty of Medicine, Zanjan University of Medical Sciences, Zanjan, Iran.; 2. Department of Clinical Psychology, Social Determinants of Health Research Center, Faculty of Medicine, Zanjan University of Medical Sciences, Zanjan, Iran.; 3. Department of Neurology, Faculty of Medicine, Vali Asr Hospital, Zanjan University of Medical Sciences, Zanjan, Iran.; 4. Department of Physiology, Faculty of Medicine, Zanjan University of Medical Sciences, Zanjan, Iran.

**Keywords:** Neurofeedback, Migraine, Sleep, tDCS

## Abstract

**Introduction:**

Migraine is considered one of the most common primary headache disorders. Migraine attacks may occur due to a lack of sleep. Furthermore, sleep is regarded as one of the smoothing factors of migraine pain. Patients with sleep disorders often suffer from headaches when they wake up compared with healthy individuals.

**Methods:**

This research was a quasi-experimental study with a pre-test-post-test design and a 2-month follow-up. The samples included 20 migraine patients within the age range of 15 to 55 years who were selected as volunteers for treatment by the neurologists and psychiatrists during 2017. The initial evaluation was then conducted based on the inclusion and exclusion criteria and using the Ahvaz migraine questionnaire, and Pittsburgh sleep quality index. The patients were randomly assigned to two neurofeedback (n=10) and transcranial Direct Current Stimulation (tDCS) (n=10) groups and evaluated three times. The obtained data were analyzed by the repeated measures ANCOVA and Chi-square test in SPSS.

**Results:**

Based on the scores of both groups, no significant difference was observed between neurofeedback and tDCS groups. However, based on the results, neurofeedback decreased sleep latency, whereas tDCS increased sleep efficiency. Overall, these two treatments were effective in improving subjective sleep quality and sleep quality.

**Conclusion:**

Both neurofeedback and tDCS treatments could significantly enhance sleep quality of the patients in the post-test and 2-month follow-up. Given the effectiveness of both treatments, neurofeedback and tDCS are recommended to be used for improving the sleep status of patients with migraine.

## Highlights

- Neurofeedback treatment improved subjective sleep quality and sleep quality
- Sleep latency decreased in the neurofeedback group.
- Transcranial direct current stimulation improved subjective sleep quality, sleep efficiency, and sleep quality.
- No significant difference was observed between neurofeedback and transcranial direct current stimulation.
- These two treatments were effective in sleep quality.

## Plain Language Summary

Migraine is a chronic and progressive neurovascular disorder of the brain, which has unfavorable effects on the patient’s life. Individuals with poor sleep quality are more likely to suffer from migraine compared with those with adequate sleep. Besides, sleep is regarded as one of the smoothing factors related to migraine pain. Therefore, considering a strong relationship between sleep disorder and migraine, the present study compared neurofeedback and transcranial Direct Current Stimulation (tDCS) treatments to identify a more effective treatment for improving the sleep quality of patients with migraine. The results indicated that the tDCS method significantly increased subjective sleep quality, sleep efficiency, and total sleep quality. Also, neurofeedback treatment increased subjective and total sleep quality, whereas it decreased sleep latency. In general, based on the findings, no significant difference was observed between two treatments regarding the components of sleep quality and total sleep quality. Therefore, both neurofeedback and tDCS treatments are effective and efficient for migraine patients with poor sleep quality.

## 1. Introduction

Headache is regarded as one of the challenges of human being health, and migraine is one of the most prevalent primary headaches. Migraine is a chronic and progressive neurovascular disorder of the brain with harmful effects on the patient’s life. Migraine headaches that are often severe, pulsating, and more unilateral, last for 2–3 days and have symptoms, such as nausea, vomiting, intolerability of the light and sound, neck pain, and muscle tension (Mottaghi, Khorvash, Askari, Iraj, & Ghiasvand, 2012).

The prevalence of migraine headaches in men and women is 4%–6% and 13%–18%, respectively (Natoli et al., 2009). Headache attacks may begin with stressful changes in life, including puberty, changing spatial and social situations, and numerous special events or experiences. Poor sleep quality is considered as one of the predictors of headache attacks (Aghayusefi & Meymand, 2013; Sullivan & Martin, 2017). Also, migraine attacks may be related to inadequate sleep. In other words, sleep is one of the factors that alleviate migraine pain.

The incidence of sleep disturbances is higher in chronic headaches. Several studies have demonstrated that compared with healthy people, patients with a sleep disorder are more likely to suffer from headaches when they are waking up. Alberti concluded that most migraine attacks are predictable considering the duration of the last night’s sleep (Asadnia, Sepehrianazar, Aghdam, & Saadatmand, 2013). The quality of sleep depends on subjective supposition about the easy onset of sleep, sleep maintenance, total sleep duration, and waking up early (Asadnia et al., 2013). Furthermore, Isik et al., indicated that the prevalence of sleep disorders was higher among children with migraine compared with their healthy counterparts (Isik et al., 2007). Similarly, Sullivan et al., found that the frequency of migraine attacks was significantly correlated with sleep duration and poor sleep quality (Sullivan & Martin, 2017). Besides, Safavi et al., observed a significant relationship between sleeping and resting patterns and migraine headaches (Safavi, Nazari, & Mahmodimajdabadi, 2008). Kelman and Rains reported that patients with migraine had difficulty in initiating and maintaining sleep (Kelman & Rains, 2005). Moreover, Isik et al., emphasized that changes in sleep pattern and quality, even though minor, could interfere with the onset of migraine headache (Sadati, Bakhteyar, Saadatmand, Saadatmand, & Asadnia, 2017).

Regarding sleep quality, Nasiri et al., demonstrated that neurofeedback therapy reduced insomnia, whereas it increased the sleep quality of patients (Basiri, Namdari, & Abedi, 2014). Additionally, Minichino et al., (2014) investigated the effectiveness of transcranial direct current stimulation (tDCS) protocol on sleep quality of bipolar patients and found a remarkable improvement in the sleep quality of these patients (Minichino et al., 2014). Likewise, Ruggiero et al., indicated that tDCS improved sleep efficiency (Ruggiero et al., 2017). Migraine imposes a high social and medical burden on the community.

Ineffective treatment of acute migraine is a significant risk factor, which exacerbates this disorder and along with increased drug consumption, leads to headache progression. Therefore, the individual becomes more sensitive in the long-term with an increase in the severity of the disorder. As a result, a safe and effective therapy with fewer side effects is required (Smitherman, 2016). Considering the short-term treatment, lack of side effects and the effects on brain waves and interactions, neurofeed-back has various advantages over many interventions, such as pharmacologic interventions.

It is a complex therapeutic system and a safe and noninvasive method, which improves brain cell growth and change. In this method, the patients are first informed about the abnormal activity of their brain waves and then are rewarded, whenever they can inhibit or strengthen the intended activities in accordance with their anomaly (Vosooghifard, Alizadeh Zarei, Nazari, & Kamali, 2013). The tDCS is another safe and non-invasive therapeutic technique for migraine, which is an appropriate alternative for pharmacotherapy and is employed to modify cerebral excitability (Utz, Dimova, Oppenländer, & Kerkhoff, 2010; Viganò et al., 2013). In addition, it is a neuronal therapeutic technique, which induces a direct and weak current to the cortical areas and facilities or inhibits spontaneous neuronal activity (Fregni et al., 2006). This current stimulates the underlying neurons by two electrodes with different poles, which normally include an anode and a cathode to different parts of the scalp. Stimulation of the cathode reduces brain excitability, whereas the anode stimulation leads to its increase (Nermasheiri, Ashrafi, Rostami, Bagherifar, & Hemmati, 2018).

Considering the high prevalence of migraine and a remarkable correlation between sleep disorder and migraine, and also finding no study on the improvement of sleep status of these patients by neurofeedback and tDCS, the present study was conducted to investigate the following objectives:

Determining the state of sleep quality in neurofeedback and tDCS groups before and after the intervention;

Identifying and comparing the changes in sleep quality in both neurofeedback and tDCS groups after the intervention.

## 2. Methods

### 2.1. Data Analysis

Descriptive statistics, such as mean, standard deviation (SD), and frequency, as well as inferential statistics, including the repeated measures ANCOVA, were used to analyze the obtained data. Quantitative analysis of the data was performed in SPSS V. 23.

### 2.2. Inclusion criteria

The inclusion criteria were meeting the diagnostic criteria of migraine based on the international classification of headache disorders (ICHD) diagnosed by the psychiatrist and neurologist through a diagnostic interview, obtaining the considered scores on a 25-item scale (Ahvaz questionnaire), having a minimum level of secondary education, being 15 to 55 years old, providing the written consent, and lacking participation in behavioral therapies, such as biofeedback, neurofeedback, or tDCS at least six months before the research.

### 2.3. Exclusion criteria

The exclusion criteria were suffering from physical (i.e., sinusitis, diabetes, history of epilepsy and brain damage) and mental illnesses associated with migraine headaches, having alcohol and drug addiction, being pregnancy, consuming oral hormones and contraceptives, having psychosis or psychotic disorders based on diagnostic interviews, carrying metal or other electrical devices in the head or having scar and scratch on the skin of the head.

### 2.4. Study instruments

The following questionnaires were used to collect the required data.

### 2.5. Ahvaz Migraine Questionnaire

Najjarian (1997) developed the Ahvaz migraine questionnaire (AMQ). The Cronbach alpha coefficient was used to assess the internal consistency of AMQ. The coefficients obtained for the whole sample (91.9), as well as the female (81.8) and male subjects (89), were satisfactory. In other words, the Cronbach alpha coefficient showed a total estimation of 0.92, indicating that the instrument enjoyed a good degree of reliability. This questionnaire was used to screen patients with migraine (Oreyzi & Darami, 2012).

### 2.6. Pittsburgh Sleep Quality Index

The Pittsburgh sleep quality index (PSQI) is a 19-item questionnaire, which evaluates sleep quality of the individuals over a 1-month time interval considering seven components: subjective sleep quality, sleep duration, sleep efficiency, sleep disturbances, using the sleeping medication, habitual sleep efficiency, and daytime dysfunction. Each subscale is scored from 0–3. High scores represent poor sleep quality, while scores of more than 5 demonstrate undesirable sleep quality and the fact that the person has severe or moderate problems at least in two or more than three components, respectively. The reliability of the questionnaire was obtained 0.816 in Asadnia et al., (2013) study, and Tehran Psychiatric Institute confirmed its validity for the Iranian population.

### 2.7. Research administration

The samples included patients referring to the neurology and psychiatry clinics of Shahid Beheshti Medical and Educational Center of Zanjan City, who had the ICHD criteria by the psychiatrists and neurologists, followed by the initial migraine assessment. The patients were randomly assigned to one of the neurofeedback or tDCS treatment groups along with the administration of the pharmacotherapy protocol. The treatment procedure was as follows:

Session 1. Diagnostic interview of migraine was conducted by the considered psychiatrist, and neurologist and the appropriate treatment relationship was established between the researcher and the patient. Then, AMQ and PSQI were administered to the patient.
Session 2. The patients were informed about the process of the formation and continuation of migraine attacks and neurofeedback treatment and the logic of therapy. Then along with pharmacotherapy, the patients were treated with neurofeedback over twenty 45-min sessions. During the neurofeedback therapy, several electrodes were connected to the cortex using a unique adhesive according to the international 10–20 system and the electrical modifications of the cortex were continuously recorded. The individuals who were in front of the computer could observe the video and recording of the waves. Whenever a person’s brain waves reached the intended conditions of the protocol, the image was enlarged and the person was scored. Accordingly, certain waves were suppressed or amplified. In other words, they learned to remain in a proper status and suppress the wave that caused the disease symptoms. Thus, the symptoms of the disease were eliminated and the patient turned into his normal health status (Ninaus et al., 2015). Neurofeedback protocol in migraine included theta suppression (4–8 Hz), and suppression (21–30 HZ), and sensory-motor amplification (SMR: sensory-motor rhythm; 12–15 Hz) in the T3 and T4 regions during 20 sessions, each of which lasted 45 min (Farahani, Tavallaie, AHMADI, & Ashtiani, 2014).

### 2.8. Process and content of tDCS treatment sessions

Session 1. A diagnostic interview of migraine was held by the considered psychiatrist and neurologist, through which an appropriate therapeutic relationship was developed between the scholar and the patient. Subsequently, the patient was surveyed through the AMQ and PSQI.
Session 2. Descriptions were provided regarding the formation and continuance of migraine attacks, the tDCS treatment, and the logic behind the treatment. Then, in addition to pharmacotherapy, the patients received tDCS treatment for 10 sessions. In each session, the patients were treated with a 2-mA current in a device with a 9-volt battery for 20 min. During these 10 sessions, the tDCS anode was placed over the Cz, whereas the cathode was placed over Oz for inhibition, which lasted for 4 weeks (Ghallagher & Kunkel, 2003). In the tDCS treatment, direct current was transferred from the electrodes (4×4.5 cm^2^) covered with a wet sponge using the serum. The size of electrodes was 4×3.5 cm^2^.

## 3. Results

Table 1 presents the demographic characteristics of the subjects.

**Table 1.** Demographic Characteristics of the Subjects

**Variables**		**No. (%)**		**Chi-Square Results P**

		**Groups**		

		**Neurofeedback**	**tDCS**	
Marital status	Single	6 (60)	3 (30)	0.185
	Married	4 (40)	7 (70)	
Occupation	Housewife	4 (40)	6 (60)	0.170
	Employed	3 (30)	4 (40)	
	Student	3 (30)	0 (0)	

The tDCS: Transcranial Direct Current Stimulation. P<0.05

As shown in Table 1, all subjects were female. The Chi-square test was used to compare the results of pre-test regarding marital status and occupation in both neurofeed-back and tDCS groups. Furthermore, the independent t-test and Kolmogorov-Smirnov test were employed to compare the age and homogeneity of variance based on Levene’s test for both groups. Based on the results of the Chi-square test, no significant difference was observed between the two groups in gender, marital status, and occupation. The Mean±SD age of the patients was 30.3±2.6 and 33.2±2.69 in the neurofeedback and tDCS groups, respectively. Comparing the mean age of the subjects using the independent t-test revealed no significant difference between the groups, and they were homogeneous in terms of this variable.

Based on Table 2, the results of the independent t-test indicated no significant difference between the groups regarding the dependent variables in the pre-test (P<0.05). Therefore, no significant difference was found between the groups in terms of sleep quality in the pre-test stage. To compare the tDCS and neurofeedback treatments, one-way ANCOVA was employed by controlling the initial differences between the groups as covariance in the pre-test. The assumptions of ANCOVA, such as homogeneity of regression slopes for interaction between pre-test and post-test, are described as follows:

**Table 2.** The results of Independent t-test regarding the sleep quality

**Components of Sleep Quality**	**Groups**		**Chi-Square Results P**	

	**Mean±SD**			

	**Neurofeedback**	**tDCS**	**Statistics T**	**P**
Subjective sleep quality	1.08±1.5	1.91±0.7	0.96	0.346
Sleep latency	0.78±2.2	0.7±2.5	0.89	0.382
Sleep duration	1.3±1.3	1.03±1.2	−0.18	0.854
Sleep efficiency	0.69±0.4	1.1±0.9	1.213	0.241
Sleep disturbances	0.84±1.4	1.08±1.5	− 0.745	0.473
Use of sleep medications	0.48±0.3	1.05±1.3	2.7	0.14
Daytime dysfunction	0.84±4.1	1.08±1.5	0.231	0.82
PSCI total score	9.6±4.7	11.3±2.9	0. 963	0. 348

The tDCS. Transcranial Direct Current Stimulation; M. Mean; SD: Standard Deviation; PSCI: Post-Stroke Cognitive Impairments

The F value for the quality of life was 5.5 (P=0.014), and Levene’s test was used to check the homogeneity of variance. Based on the results, Levene’s statistic was obtained 3.745 (P=0.069) for sleep quality.

Based on Table 3, no significant differences were observed between the two treatment groups regarding the components of sleep quality index and sleep quality in post-test and follow-up stages. Also, the mean score of sleep quality in the post-test stage in the tDCS group was higher than that of the neurofeedback group. However, it was higher in the neurofeedback group during the two-month follow-up compared with the tDCS group.

**Table 3.** Mean±SD and analysis of covariance statistics for comparison of neurofeedback and tDCS groups regarding sleep quality

**Variable**	**Stage**	**Groups**		**Mean Square**	**F**	**P**	**Eta**

		**Mean±SD**					

		**Neurofeedback**	**tDCS**				
Subjective sleepquality	Post-test	0.48±0.7	0.87±1.1	1.36	3.19	0.092	0.158
	Two-month follow-up	0.51±0.6	0.91±0.8	0.656	1.55	0.229	0.084
Sleeplatency	Post-test	0.99±1.9	0.96±1.4	0.844	0.865	0.365	0.048
	Two-month follow-up	1.05±1.7	0.8±1	1.23	1.75	0.203	0.094
Sleep duration	Post-test	0.42±0.2	0.7±0.5	0.392	1.3	0.2	0.071
	Two-month follow-up	0.96±0.6	1.05±0.7	0.037	0.035	0.855	0.002
Sleepefficiency	Post-test	0.1±0.1	0.42±0.2	0.265	2.98	0.1	0.149
	Two-month follow-up	0.42±0.2	0.1±0.1	0.076	1	0.33	0.5
Sleep disturbances	Post-test	0.63±1.8	0.48±1.3	1.24	3.71	0.071	0.179
	Two-month follow-up	0.52±1.5	0.51±1.4	0.061	0.213	0.65	0.012
Use of sleepmedications	Post-test	0.96±0.6	0.67±0.3	0.316	0.66	0.428	0.037
	Two-month follow-up	1.25±0.7	0.67±0.3	1.208	3.2	0.091	0.159
Daytimedysfunction	Post-test	0.99±0.9	0.87±0.1	0.264	0.315	0.582	0.018
	Two-month follow-up	1.07±0.6	0.99±1.1	1.612	2.417	0.138	0.124
PSQI total score	Post-test	2.07±6.1	2.4±5.9	0.549	0.157	0.696	0.009
	Two-month follow-up	1.4±5.9	5.3±2.5	0.016	0.008	0.93	0.000

The tDCS. Transcranial Direct Current Stimulation; M. Mean; SD. Standard Deviation; PSQI. Pittsburgh Sleep Quality Index

Considering that the within-group difference in the tDCS group was significant in sleep quality, pairwise comparisons were required to determine which sets of scores were different. To this end, the least significant difference (LSD) test was used. The findings demonstrated that tDCS treatment could significantly improve sleep quality from pre-test to post-test and then during a two-month follow-up (Table 4).

**Table 4.** Paired t-test results in a three-time evaluation of dependent variables in both groups (Least Significant Difference (LSD))

**Dependent Variable**	**Source of Differences**	**Df**	**Mean Square**	**F**	**P**	**Eta**
Subjective sleep quality neurofeedback	Within subjects	2	1.23	6.28	0.009	0.41
	Error	18				
Subjective sleep	Within subjects	2	5.23	12.5	0.000	0.581
quality tDCS	Error	18				
Sleeplatency neurofeedback	Within subjects	1.5	4.86	7.78	0.008	0.467
	Error	13.8				
Sleeplatency tDCS	Within subjects	2	1.73	2.48	0.111	0.217
	Error	18				
Sleep duration neurofeedback	Within subjects	2	1.5	1.77	0.213	0.165
	Error	18				
Sleep duration tDCS	Within subjects	2	0.433	0.854	0.442	0.087
	Error	18				
Sleepefficiency neurofeedback	Within subjects	2	4	2.25	0.134	0.2
	Error	18				
Sleepefficiency tDCS	Within subjects	1.3	0.341	5.84	0.026	0.394
	Error	11.7				
Sleep disturbancesneuro feedback	Within subjects	2	4.43	2.87	0.083	0.242
	Error	18				
Sleep disturbancestDCS	Within subjects	2	0.633	4.171	0.032	0.317
	Error	18				
Use of sleepmedications neurofeedback	Within subjects	2	0	0	1	0
	Error	18				
Use of sleepmedications tDCS	Within subjects	2	1.433	3.38	0.06	0.269
	Error	18				
Daytimedysfunctionneuro feedback	Within subjects	2	3	0.73	0.496	0.075
	Error	18				
Daytimedysfunction tDCS	Within subjects	2	2.1	2.498	0.11	0.217
	Error	18				
PSQI total scoreneurofeed	Within subjects	1.09	99	16.77	0.002	0.651
back	Error	9.841				
PSQI total scoretDCS	Within subjects	1.23	151.4	29.9	0.000	0.75
	Error	11.13				

The tDCS. Transcranial Direct Current Stimulation; M. Mean; SD. Standard Deviation; PSQI. Pittsburgh Sleep Quality Index

Based on the results, neurofeedback treatment significantly improved subjective sleep quality and sleep quality from compared with the pre-test and over a two-month follow-up. Furthermore, sleep latency decreased in the neurofeedback group. The findings provided in Table 5 indicate that tDCS treatment significantly improved subjective sleep quality, sleep efficiency, and sleep quality compared with the pre-test and during a two-month follow-up.

**Table 5.** Investigating the within-subject effect in three measurement stages among the neurofeedback and tDCS groups

**Components of Sleep Quality**	**Duration**		**Mean Difference**	**Std. Error**	**P**
Subjective Sleep quality (Neurofeedback)	Pre-test	Post-test	0.3	0.485	0.037
	Pre-test	Two-month follow-up	0.5	0.2	0.01
	Post-test	Two-month follow-up	0.2	0.554	0.193
Sleep efficiency (Neurofeedback)	Pre-test	Post-test	0.8	0.389	0.04
	Pre-test	Two-month follow-up	1.2	0.2	0.000
	Post-test	Two-month follow-up	0.4	0.3	0.2
Sleep quality (Neurofeedback)	Pre-test	Post-test	3.7	0.932	0.003
	Pre-test	Two-month follow-up	3.4	1.01	0.002
	Post-test	Two-month follow-up	0.6	0.0221	0.024
Subjective Sleep quality(tDCS)	Pre-test	Post-test	1.2	0.359	0.009
	Pre-test	Two-month follow-up	1.3	0.3	0.002
	Post-test	Two-month follow-up	0.1	1.8	0.591
Sleep efficiency (tDCS)	Pre-test	Post-test	0.9	0.348	0.029
	Pre-test	Two-month follow-up	0.7	0.3	0.045
	Post-test	Two-month follow-up	−0.2	0.2	0.168
Sleep quality (tDCS)	Pre-test	Post-test	6.2	1.12	0.001
	Pre-test	Two-month follow-up	5.4	0.73	0.000
	Post-test	Two-month follow-up	0.2	0.53	0.716

## 4. Discussion

There is a complex and multidimensional relationship between sleep and headache, and also, the headache may be one of the underlying symptoms of sleep. Moreover, sleep disorder may lead to a headache. Additionally, both sleep disturbances and headache are probably the signs of an underlying illness. A headache at night or immediately after waking up can be regarded as a sign of sleep disorder (Annarumma, D’Atri, Alfonsi, & De Gennaro, 2018). In Sullivan & Martin (2017) study, it was concluded that the frequency of migraine attacks has a significant correlation with sleep duration and poor sleep quality. Migraine is one of the most common types of headaches that affects all aspects of the individual and social life of the people and also their working life (Safavi et al, 2008). Migraine is accompanied by nausea, vomiting, light and voice intolerance, neck pain, and muscle tension (Mottaghi et al., 2012). Therefore, these patients need an effective and efficient treatment to improve their pain and sleep conditions.

In this respect, neurofeedback and tDCS are two effective therapies. The neurofeedback is used to assess the alterations of the brain states and can modify, strengthen, and enhance the efficie ncy of the brain cells. As a result, sleep pattern alteration and regulation are among the first changes that the patients typically observe after initiating the neurofeedback treatment (Basiri et al., 2014). Frass et al., (2016) found that tDCS treatment reduced sleep duration in healthy individuals. Evaluating the scores of both groups revealed that tDCS and neurofeedback protocols along with pharmacotherapy, were effective in improving subjective sleep quality and sleep quality. In other words, there was no significant difference between these two treatments. However, neurofeedback was found to reduce sleep latency, whereas tDCS increased sleep efficiency (Frass et al., 2016).

Sterman et al., concluded that neurofeedback training (12–14 Hz, SMR) on cats changed their sleep Electroencephalogram (EEG). The beta wave amplitude (15–30 Hz) is a prominent feature of the EEG during awakening and increases the cortical stimulation (Dowom, Roshanaei, & Darvishi, 2015). As neurofeedback corrects the abnormal brain waves, an increase in the SMR wave leads to the modification of high beta waves to SMR, leading to facilitating sleep and reducing sleep latency. In addition, Najafabadi et al., (2014) indicated that neurofeedback reduced anxiety by increasing the SMR and decreasing the theta frequency (Najafabadi, Salehi, Rahmani, & Imani, 2014). Migraine is accompanied by anxiety (Leahy, Holland, & McGinn, 2011). Neurofeed-back helps the individual to safely control his psychological state and deal with anxious thoughts throughout his daily life (Najafabadi et al., 2014). The protocol used in this study on patients with migraine decreased their anxiety. Furthermore, a decline in anxiety can lead to an increase in SMR, while resulting in a decrease in theta and high beta frequency, improving sleep quality and, consequently, subjective sleep quality.

Erwin underlined that physiological and pathological factors, such as pain and discomfort, influence the quality of sleep and make it difficult for the patient to fall into a deep sleep (Lee & Douglass, 2010). The higher is the depth of the sleep; the better would be its quality (Harvey, Stinson, Whitaker, Moskovitz, & Virk, 2008). Reducing the pain and improving the headache status in tDCS treatment can lead to a night of deep sleep and improve the efficiency and quality of sleep in this group. The results of the present study are in line with the findings of Ruggiero et al., (2017) who indicated that the sleep efficiency increased by tDCS treatment.

The data collection tool was a questionnaire that made this research subjective. Using other tools such as actigraphy and or polysomnography could make it more objective. The lack of polysomnography was another limitation of the present study, which is recommended to be used in future studies. Besides, the role of lifestyle in sleep status was not investigated in this research.

The results of the study may not be generalized to other subjects because of a small sample size of only 20 patients who referred to the Shaheed Beheshti Hospital of Zanjan. Implementing relevant studies using a larger sample size will increase the generalization of these findings. Furthermore, it is suggested that the tDCS and neurofeedback treatment protocols be conducted based on the brain map of each individual and Quantitative Electroencephalography (QEEG) to identify and treat the precise involved brain regions.

## 5. Conclusion

The results should be interpreted with caution. Although the researchers attempted their best to manage the conditions, therapeutic situations, such as psychological treatment on human subjects, are difficult to control. Generally speaking, both neurofeedback and tDCS treatment protocols along with pharmacotherapy significantly improved the sleep quality of the patients in the post-test compared with the pre-test and during a 2-month follow-up. Considering the effectiveness of both treatments, we recommend this intervention for improving the sleep status of migraine patients.

## Ethical Considerations

### Compliance with ethical guidelines

This clinical trial was approved by the Ethics Committee of Zanjan University of Medical Sciences (Approval Code No.: ZUMS.REC.1396.152) and registered at the Iranian Registry of Clinical Trials (IRCT20171023036952N1). The study population included all the patients with migraine in Zanjan City, Iran. A total of 20 women with migraine were selected using a purposive sampling technique and randomly assigned into two groups.
